# Experimental study on the optimization of ANM33 release in foam cells

**DOI:** 10.1515/biol-2022-0564

**Published:** 2023-02-23

**Authors:** Chen Yuan, Liyun Liu, Baihetiya Tayier, Ting Ma, Lina Guan, Yuming Mu, Yanhong Li

**Affiliations:** Department of Echocardiography, First Affiliated Hospital of Xinjiang Medical University, State Key Laboratory of Pathogenesis, Prevention and Treatment of High Incidence Diseases in Central Asia, Urumqi, China; Xinjiang Key Laboratory of Ultrasound Medicine, Urumqi, Xinjiang 830011, China

**Keywords:** drug delivery, dual targeting, foam cells, atherosclerosis

## Abstract

Given the miR-33’s mechanistic relationships with multiple etiological factors in the pathogenesis of atherosclerosis (AS), we investigated the therapeutic potentials of dual-targeted microbubbles (HA-PANBs) in foam cell-specific release of anti-miR-33 (ANM33) oligonucleotides, resulting in the early prevention of AS progression and severity. The intracellular localization, loading optimization, and therapeutic effects of HA-PANBs were examined in detail in a co-cultured cell model of phagocytosis. Compared with non-targeting nanobubbles (NBs) and single-targeted microbubbles as controls, HA-PANBs efficiently delivered the ANM33 specifically to foam cells via sustained release, exhibiting its clinical value in mediating RNA silencing. Moreover, when used at a dose of 12 µg/mL HA-PANBs per 10^7^ cells for 48 h, a higher release rate and drug efficacy were observed. Therefore, HA-PANBs, effectively targeting early AS foam cells, may represent a novel and optimal gene therapy approach for AS management.

## 1 Introduction

AS is a chronic inflammatory vascular disease [1,2,3] that jeopardizes the quality of life and has become a major public health problem worldwide. Prevention and treatment of AS are urgently needed. Local inflammatory responses, particularly macrophages (Mφ), are considered to be one of the key factors leading to the occurrence and development of AS [4,5,6]. Early Mφ can delay the disease progression by disposal of cholesterol deposited in the atherosclerotic plaque (cholesterol efflux) [7,8,9,10]. When the oxidized low-density lipoprotein (ox-LDL) continues to accumulate in Mφ, macrophage-derived foam cell formation occurs, leading to early atherogenesis and disease progression. Recent studies have shown that miR-33 in Mφ accelerates the impairment of cholesterol efflux and that anti-miR-33 (ANM33) effectively reduces the inflammatory response within the plaque. However, a non-selective inhibition of miR-33 in systemic Mφ can lead to severe side effects [11,12].

The development of AS involves a series of cellular and molecular events. Ultrasound-based molecular imaging for targeted detection of cardiovascular disease-associated molecular markers and genetic signatures plays crucial roles in the diagnosis and therapy of adverse cardiovascular events [13]. Therefore, precise targeting of the damaged foam cells in AS plaques may provide new treatment possibilities. Ideally, a targeting agent should reach its destination with high specificity, but a wide distribution of immune Mφ cells throughout the body poses a great challenge to the targeting agents’ degree of specificity. Studies have shown that a two-stage targeting design and HA-coating of microbubbles effectively shield them from binding to off-target liver receptors, thereby enhancing their targeting efficiencies [14–16].

Based on the above findings, we have successfully constructed a dual-targeting ultrasound microbubble contrast agent, HA-PANB, with varying conjugation methods for a non-invasive assessment of early AS [17]. However, certain technical challenges are still unmet, particularly regarding the loading of ANM33 into microbubbles and their engulfment by Mφ through phagocytosis. We believe that a thorough investigation of the optimal dose and treatment conditions for HA-PANBs is the key to further improvement of its therapeutic efficacy, in addition to a better clinical value.

## 2 Materials and methods

### 2.1 Materials

Human umbilical vein endothelial HUVEC-T1 cells (Procell Life Science & Technology, China), RAW264.7-T1 Mφ (Procell Life Science & Technology, China), oxidized low-density lipoprotein (ox-LDL; Yiyuan Biotechnology, China), tumor necrosis factor-α (TNF-α; Bioss, China), and Transwell chambers (Corning, USA) were used. FITC-labelled anti-human CD44 flow cytometry antibody (Proteintech, USA), high-glucose DMEM (ScienCell, China), CCK-8 kit (Tongren Chemical, Japan), fetal bovine serum (FBS; ScienCell, USA, Israel), and double antibody (Gibco, USA) were purchased for this study. Endothelial cell basal medium (ECM; ScienCell, USA), FBS (ScienCell, USA), endothelial cell growth factor (ScienCell, USA), double antibodies (ScienCell, USA), and Fluorescence microscope (LEICA CTR6000, Germany); laser confocal microscope (Leica SP8, Germany); flow cytometer (Beckman, USA); laser nanoparticle size potential analyzer (Malvern, UK) were used.

### 2.2 Preparation of targeted ultrasound microbubbles

Carrier core nanobubbles (NBs) were prepared by thin film hydration, and microbubbles loaded with PM1 (PCNBs) were prepared by grafting DSPE-PEG2000-maleimide-PM1 onto the NB surface. ANM33 was connected via electrostatic adsorption and covalent bonding, and hyaluronic acid (HA) was covalently connected. PM1 and HA were the targets, and ANM33 was the intervention drug.

### 2.3 Establishment of *in vitro* co-culture system using damaged endothelial cells and Mφ (HUVEC/MΦ)

#### 2.3.1 Culture of multilayer cells

**Figure 1:**
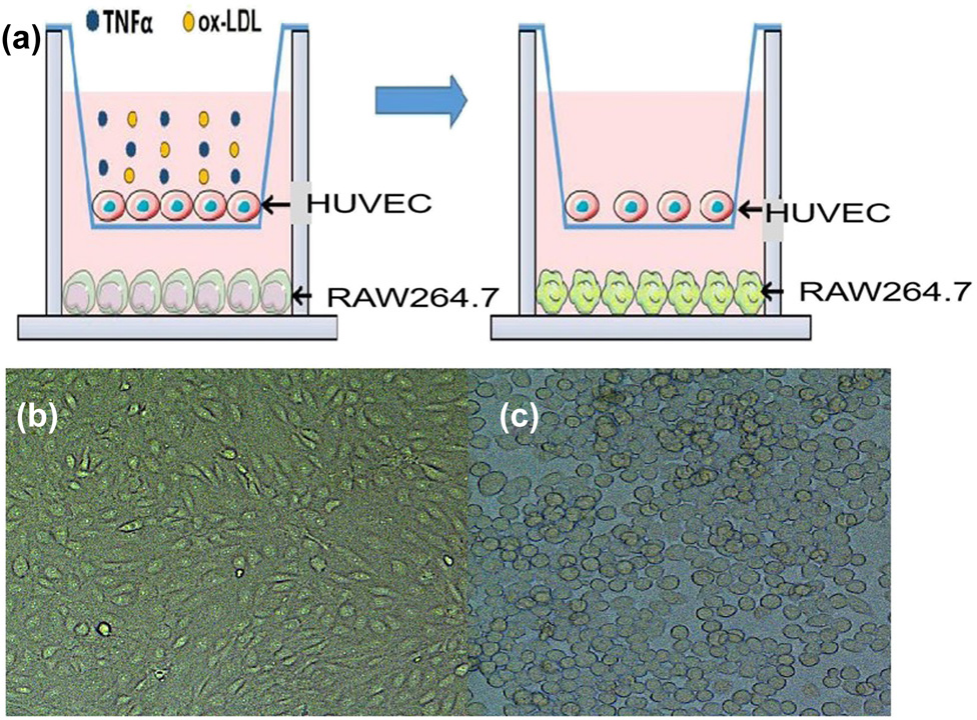
Establishment of injured endothelial-macrophage co-culture system. (a) Schematic diagram of co-culture system; (b) upper HUVECs; and (c) lower macrophage MΦ.

The specific steps for simulating damaged blood vessels in the body are shown in Figure 1. HUVECs were first inoculated on a gelatin-coated Transwell polycarbonate filter membrane and cultured for 48 h to form an endothelial monolayer. Then, MΦs were seeded in the lower chamber of the Transwell and co-cultured with HUVECs in the upper chamber for 24 h, and the medium was replaced with M199 medium containing TNF-α and ox-LDL for 24 h. The inoculation density of HUVECs was 5.0 × 10^4^/mL, and the inoculation density of MΦs was 2.0 × 10^4^/mL.

Cells were monitored under a light microscope for the construction of a growth curve. This experiment was independently repeated for at least three times. When cells reached confluency, they are either passaged or used for the experiment.

#### 2.3.2 Construction and identification of damaged HUVECs

After cell inoculation, the density reached 70–80% after 2 days of culture. According to the instructions, 5 µL of fluorescent CD44 antibody was added to 3.0 × 10^6^ cells, mixed and incubated at 4°C in the dark for 30 m, the fluorescence intensity on the HUVEC surface was analyzed by flow cytometry [18] in at least three independent repetitions. HUVECs in the HUVEC/MΦ system without TNF-α and ox-LDL treatment were collected as the control group.

#### 2.3.3 Influence of different treatments on MΦ cell viability

This group of experiments was performed in 96-well plates to seed only a single layer of MΦ cell suspension (100 µL/well). After the cells were allowed to adhere to the wall for 4 h, the medium was replaced with fresh medium containing different concentrations of TNF-α and ox-LDL, and the culture plate was placed in an incubator for pre-culture (37°C, 5% CO_2_) for 24 h. Next 10 µL of CCK-8 solution was added to each well. The culture plate was incubated in an incubator for 1–4 h, and the absorbance at 450 nm was measured with a microplate reader and analyzed.

A CCK-8 kit was used to evaluate the cytotoxicity of the HA-PANBs. RAW264.7 cells were cultured by adding 100 µL of cell suspension to a 96-well plate and preculturing the plate in an incubator for 24 h (37°C, 5% CO_2_). Then, 10 µL of HA-PANBs at concentrations of 10^8^, 10^7^, 10^6^, 10^5^, or 10^4^ was added to the culture plate, which was incubated for 24 and 48 h. Ten microliters of CCK solution was added to each well, and the culture plate was incubated in the incubator for 1–4 h. The absorbance was measured at 450 nm with a microplate reader. The assay was repeated in at least three biological replicates.

#### 2.3.4 HUVEC cell layer permeability test

FITC labeled albumin was added to the Transwell upper chamber. After 1 h, the fluorescence intensity of the upper chamber and the lower chamber were measured with a fluorometer and repeated the above operation at least three times. Permeability (*P*) = (*V*_L_ × *C*_L_)/(*V*_U_ × *C*_U_) × 100%; (*C*_L_ and *C*_U_ are the concentrations of FITC labeled albumin in the lower and upper chambers, respectively, and *V*_L_ and *V*_U_ are the medium volumes of the lower and upper chambers, respectively).

### 2.4 Identification of foam cells

#### 2.4.1 Cholesterol quantification

A cholesterol quantification kit was used to observe the changes in the amount of cholesterol in Mφ after incubation with different concentrations of ox-LDL. After inducing the cells with ox-LDL at concentrations of 0 mg/L, 10 mg/L, 20 mg/L, 40 mg/L, 60 mg/L, and 80 mg/L, the total cholesterol (TC), free cholesterol (FC), and cholesterol ester (CE) contents and specific gravity of the cells were observed, and the proportion of CE was calculated as (TC – FC)/TC × 100% and the above operation was repeated for at least three times.

### 2.5 Evaluation of targeting *in vitro*

The fluorescent carbocyanine dye DiI was conjugated to various microbubbles and diluted with fresh serum medium and added to the upper chamber of the injured co-culture system when the cell count reaches to 10^7^ observed with the microscope, followed by incubation for 30 min. This operation was repeated for at least three times.

#### 2.5.1 Experimental grouping and intervention methods

The experimental grouping and intervention methods are given in Table 1.

**Table 1:** Experimental grouping

Group	Intervention method	Remarks
PBS	PBS + cell model	Blank
NBs	NBs + cell model	Non-targeted NBs
PNBs	PNBs + cell model	Single targeted NBs surface carrying PM1
HA-NBs	HA-NBs + cell model	Single targeted NBs surface carrying HA
HA-PNBs	HA-PNBs + cell model	Dual-targeted NBs, surface carrying HA PM1

#### 2.5.2 Standing/shaking co-incubation method

Before the experiment, hyaluronidase (HAase; 2 mg/mL) was added to the lower chamber of the Transwell. Various NBs were then added to the double-layer co-culture system of injured endothelial-Mφ, incubated for 30 min, and rinsed three times with PBS. The homing effect of HA-PANBs was evaluated by the following detection methods: *in vivo* imaging system (IVIS) for the DiI fluorescent intensity in the lower chamber of the Transwell, confocal microscopy for the number of DiI in the lower chamber with respect to the underlying Mφ, and flow cytometry for the amount of DiI in the lower chamber [18]. The whole process was repeated at least three times for statistical validation.

### 2.6 Adhesion and release time optimization

Cells were plated at the upper chamber and pretreated with 2 mg/mL HAase, followed by the addition of AF-488 and Cy3 double fluorescently labeled HA-PANBs 10^4^, 10^5^, 10^6^, 10^7^, and 10^8^ were added, respectively, for incubation of 2 h, 6 h, 12 h, 24 h, and 48 h. The loading dose of ANM33 into HA-PANBs was optimized at 12 µg/mL [17]. The above process was repeated at least three times. The same volume of DMEM containing 20% (v/v) FBS was added to all wells the next day, and the localization of lipid contrast agent particles in the MΦ was examined by confocal scanning microscopy. In the control group, normal HUVEC/MΦ cells that were not induced by injury were selected.

### 2.7 *In vitro* release of microbubbles

Different microbubbles with and without 2 mg/mL HAase were sealed in a dialysis bag (MW cutoff = 8–12 kDa) in 200 mL of release buffer (0.05% sodium dodecyl sulfate in PBS, pH 7.4), and stirred gently at 37°C. An equal volume (0.5 mL) of release buffer was collected and replenished at each time point to allow a total reaction time of 72 h. The ANM33 concentration in the release buffer collected at each time point was measured using a multi-function microplate reader repeatedly at least three times. As a control, the release of ANM33 from microbubbles in each group in the absence of HAase was also determined. Experimental grouping and intervention methods are shown in Table 1.

### 2.8 Fluorescence quantitative PCR

The RNA concentration and purity were detected by Nanodrop2000 after total RNA extraction. After reverse transcription reaction, Real-Time PCR was carried out for 40 cycles with melt curve analysis. GAPDH was used as the internal reference gene for normalization. The 1/2^△△Ct^ value was used for calculation and analysis and the assay was repeated at least three times. The primer sequences were synthesized and purified by Wuhan Sevier Biotechnology Co, Ltd. The primer sequences are shown in Table 2.

**Table 2:** RT-PCR primer sequences

Amplified gene		Primer sequence (5′–3′)
miR33	1	CTCAACTGGTGTCGTGGAGTCGGCAATTCAGTTGAGGTGATGCA
	2	ACACTCCAGCTGGGCAATGTTTCCACAGTG

### 2.9 Statistical methods

Measurement data that conformed to a normal distribution are represented as the “mean value ± standard deviation,” while data that did not conform to a normal distribution are represented as the "median and interquartile range." Statistical analyses mainly consisted of one-way analysis of variance (ANOVA) and independent sample *t* tests. When the data did not conform to a normal distribution, a nonparametric test was used; repeated measurement data analysis used repeated measurement analysis of variance. The confidence interval for determining the differences between groups was set to 95%, and it was considered statistically significant when *P* < 0.05.

## 3 Results

### 3.1 Characterization of HA-PANBs

**Figure 2:**
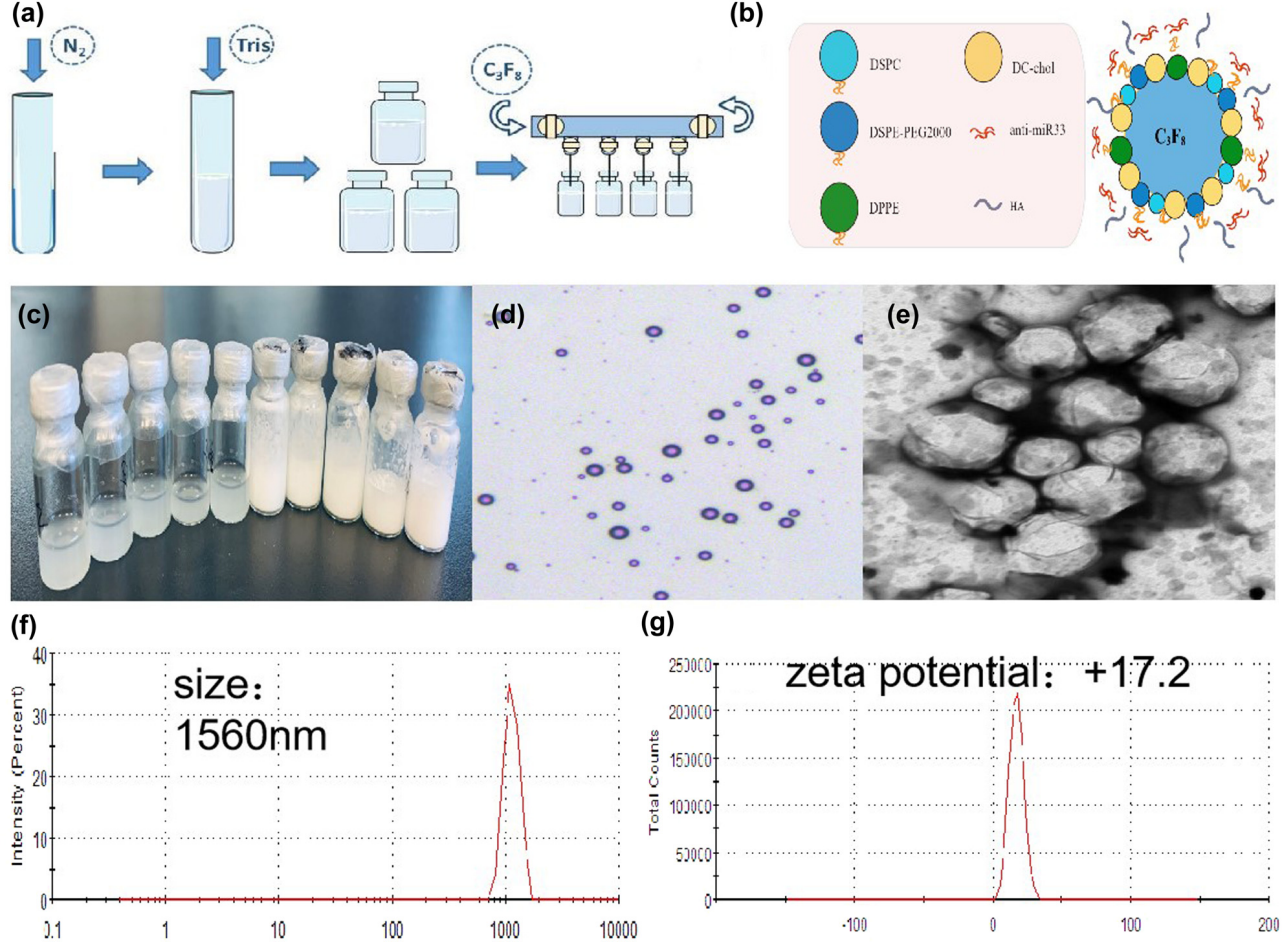
The morphology and structure of the NBs. (a) Preparation of NBs; (b) preparation of HA-PANBs; (c) the phospholipid suspension before and after mechanical vibration; (d) photomicrograph of NBs; (e) TEM image of HA-PANBs; (f) size of HA-PANBs; (g) zeta potential of HA-PANBs.

Morphology, particle size, size disruption, zeta potential of HA-PANBs were assessed as shown in Figure 2.

### 3.2 Identification of damaged HUVECs

**Figure 3:**
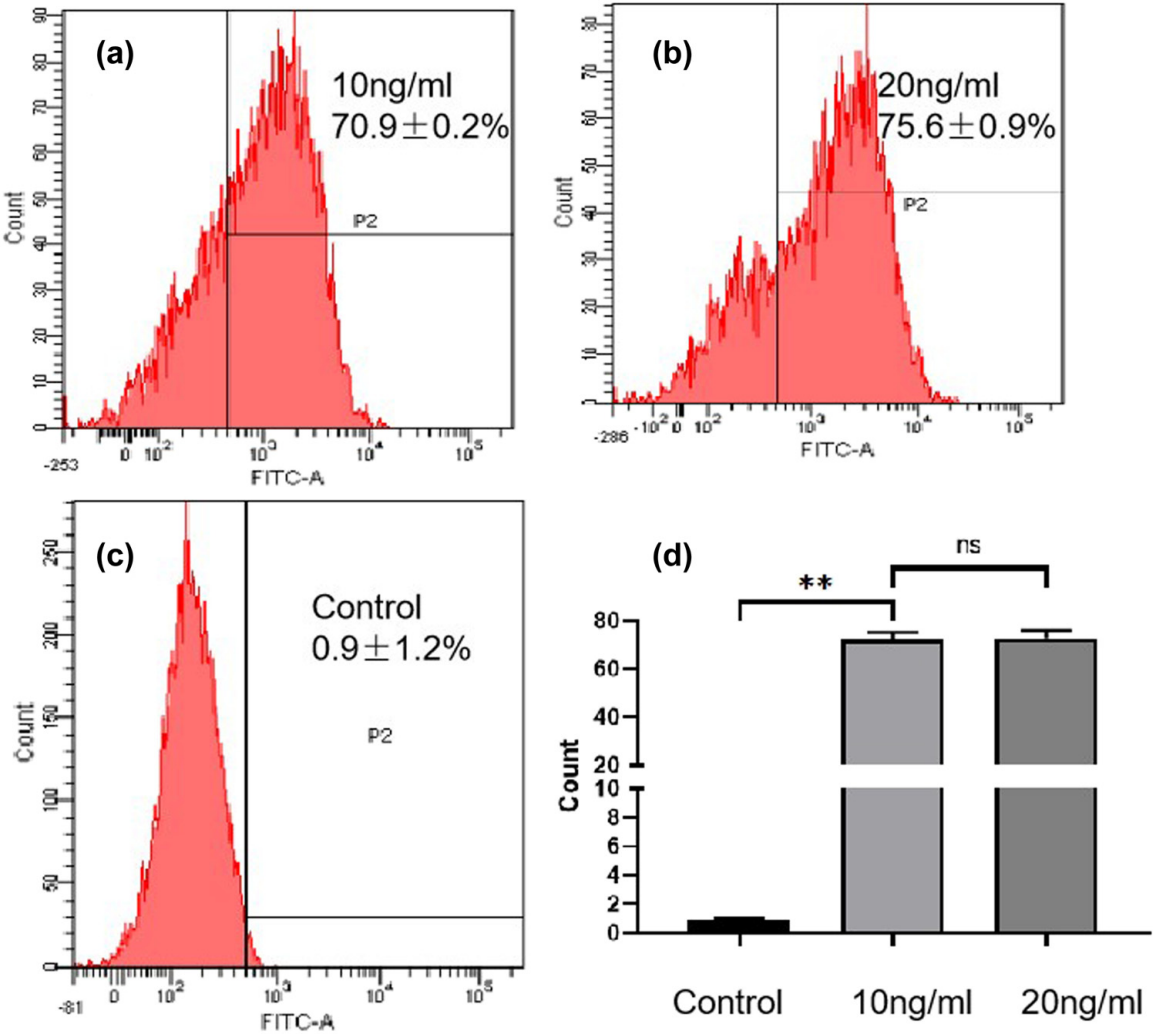
Identification of damaged HUVECs. (a) Treated with 10 ng/mL TNF-α and ox-LDL; (b) treated with 20 ng/mL TNF-α and ox-LDL; (c) control group; (d) quantitative results showing that the model significantly expressed CD44 antibody; **: *P* < 0.01.

The results of flow cytometry showed that the proportion of HUVECs expressing CD44 was 70.9 ± 0.2% after treatment with 10 ng/mL TNF-α, while the proportion in the 20 ng/mL TNF-α group was 75.6 ± 0.9%. There was no significant difference between the experimental groups (*P* > 0.05), while both the experimental groups compared with the blank control had differences (*P* < 0.05), as shown in Figure 3.

### 3.3 Identification of foam cells

**Figure 4:**
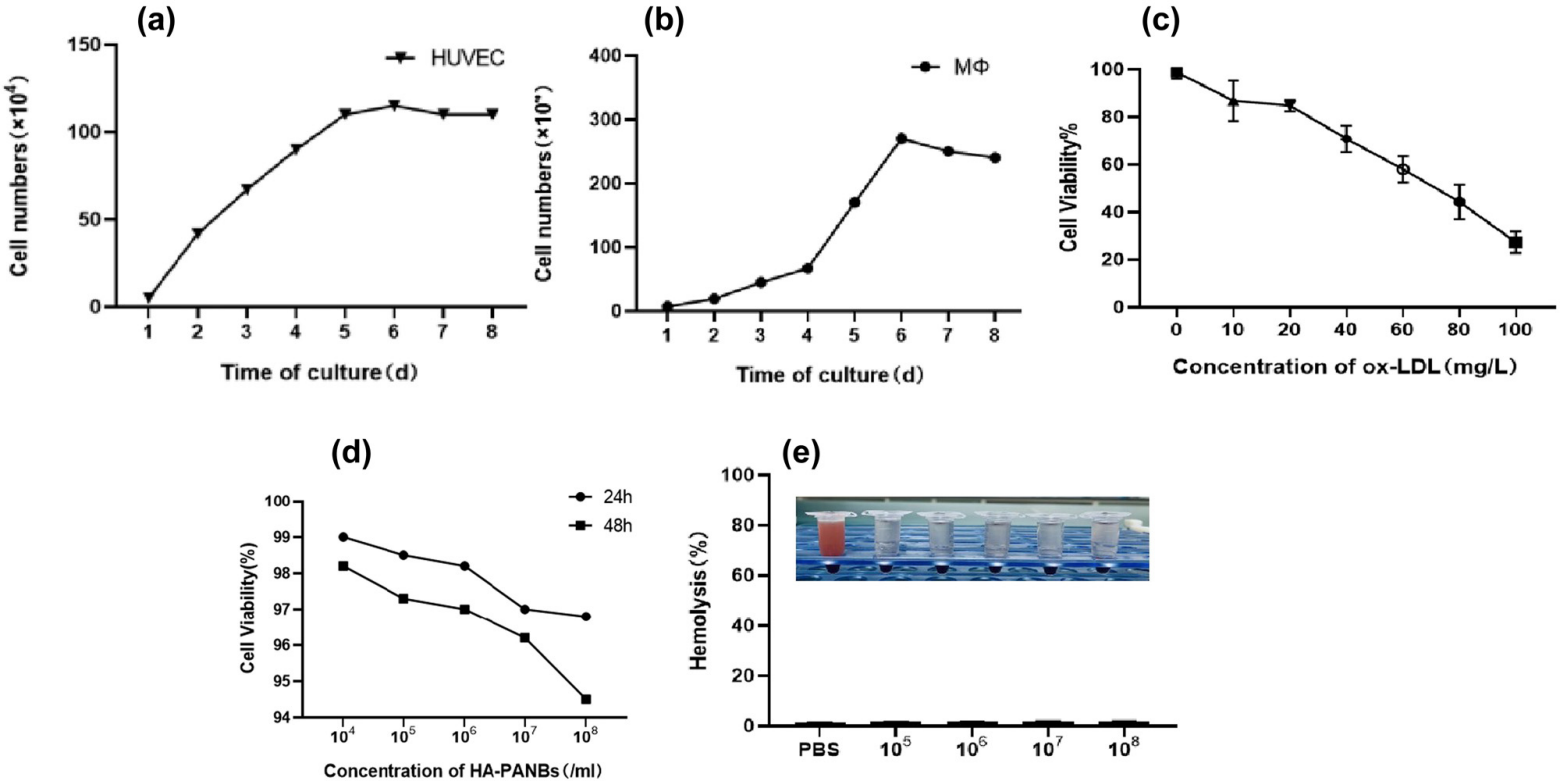
Cell growth curve and cell viability after intervention. (a) Endothelial cell growth curve; (b) macrophage growth curve; (c) macrophage activity after ox-LDL intervention; (d) cytotoxicity changes over time; and (e) *in vitro* hemolysis test.

The results showed that the TC and CE contents in the cell and the specific gravity increased in a concentration-dependent manner, and the degree of foam cell formation gradually increased, when the concentration of ox-LDL exceeded 60 mg/L, the intracellular cholesteryl ester specific gravity exceeded 50%, as shown in Table 3. At the same time, as the concentration of treatment drugs increased, MΦ activity decreased, as shown in Figure 4.

**Table 3:** Specific gravity of cholesteryl esters in different degrees of lipid-bearing cells

ox-LDL (mg/L)	TC (µmol/L)	FC (µmol/L)	CE (µmol/L)	CE/TC (%)
0	36.54 ± 1.1	26.45 ± 1.8	10.09 ± 1.1	28 ± 1.3
10	41.55 ± 3.8	24.63 ± 2.6	16.92 ± 1.2	41 ± 2.1
20	48.33 ± 5.5	26.49 ± 3.7	21.84 ± 1.2	45 ± 2.6
40	76.12 ± 6.1	37.65 ± 3.9	38.47 ± 4.2	51 ± 4.8
60	93.9 ± 6.7	37.79 ± 4.5	56.12 ± 5.5	60 ± 5.1
80	113.94 ± 3.9	34.7 ± 6.3	79.25 ± 4.3	70 ± 3.9

### 3.4 Effect of microvesicles on cell viability

Mφ reached a saturation density of 2.7 × 10^6^ on the sixth day. The endothelial cells reached a saturation density of 1.1 × 10^6^ on the fifth day (Figure 4). The results of the biosafety evaluation of HA-PANBs showed that when the concentration of ox-LDL was gradually increased, the cell viability decreased accordingly. In addition, the solutions containing HA-PANBs at different concentrations were clear (Figure 4e).

### 3.5 Permeability test of HUVEC cell layer

**Figure 5:**
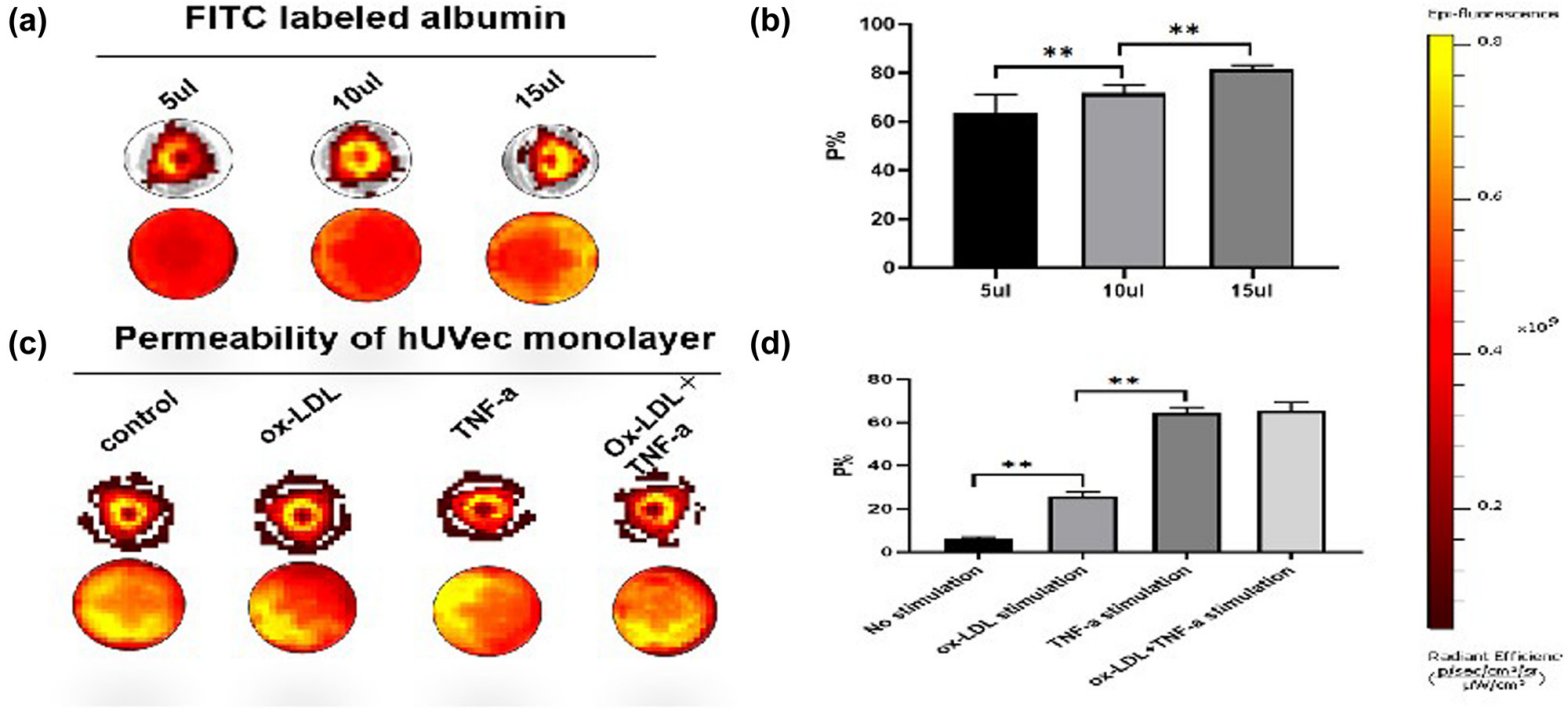
HUVEC cell layer permeability assay. (a) Estimation of endothelial layer permeability after addition of different doses of albumin; (b) quantitation of endothelial layer permeability; (c) effect of different intervention methods on endothelial layer permeability; and (d) quantitative comparisons among different interventional approaches; **P < 0.01.

After adding 5, 10, and 15 µL FITC labeled albumin, respectively, the fluorescence intensity of the lower macrophage layer can reach more than 50% as shown in Figure 5. When measuring the permeability, it can be found that there is no significant increase in the permeability of the endothelial cell layer without intervention or only adding ox-LDL, but higher permeability can be achieved only by adding TNF-α and ox-LDL group, that is, more than 60%.

### 3.6 *In vitro* targeting situation

**Figure 6:**
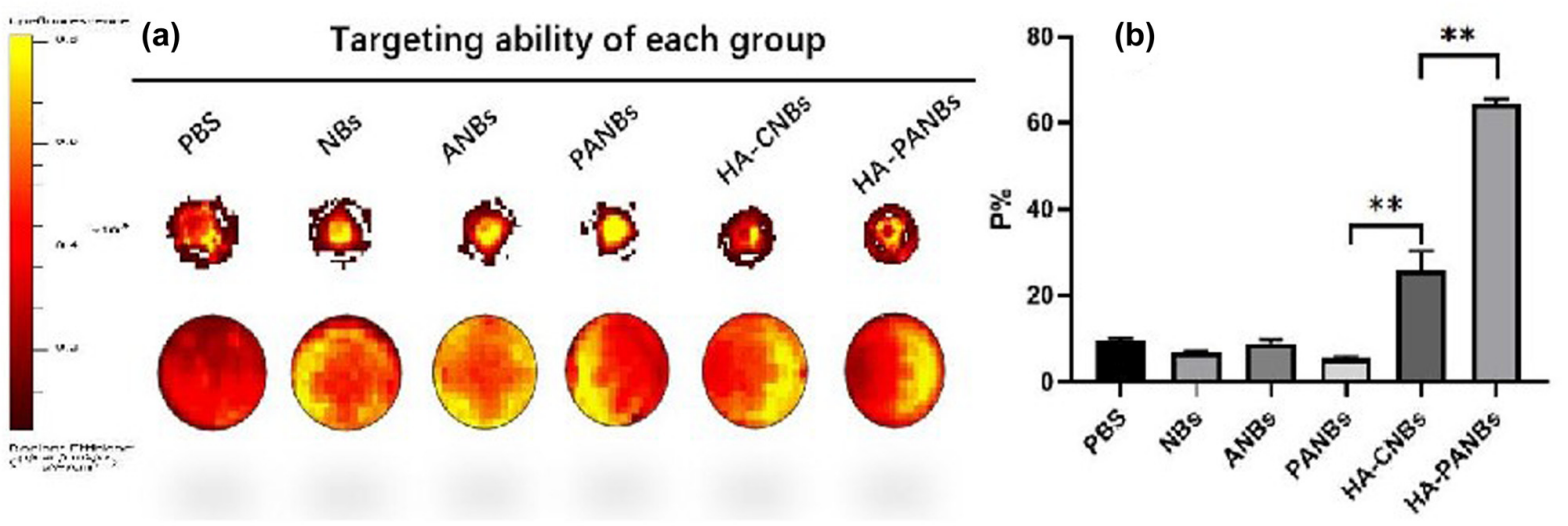
Observation of *in vitro* targeting by small animal imager. (a) Targeting situation of each group after intervention in foam cell bilayer model and (b) quantitative results; **: *P* < 0.01.

When using IVIS to detect the fluorescence intensity of DiI in the lower chamber of the Transwell, the targeting efficiencies are as follows: 9.57 ± 0.7% for PBS, 6.935 ± 1.1% for NBs, 9.27 ± 1.3% for ANBs, 9.27 ± 1.3% for PANBs, 23.055 ± 3.5% for HA-CBNs, and 64.945 ± 1.2% for HA-PANBs (as shown in Figure 6).

### 3.7 Optimization of adhesion and transfection conditions

**Figure A1:**
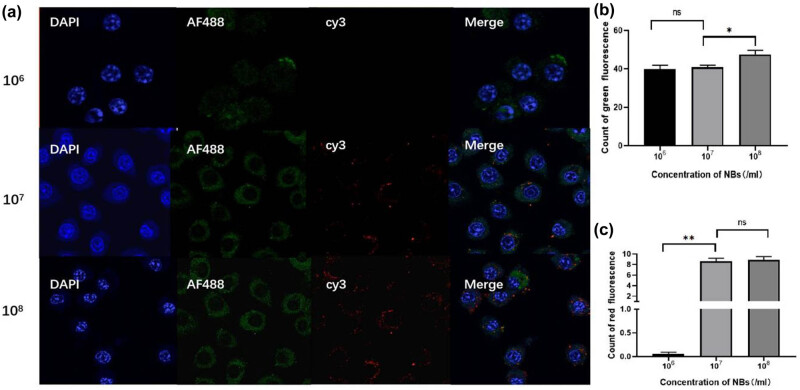
Laser confocal microscope observation of *in vitro* targeting of HA-PANBs with different concentrations. (a) Laser confocal images after different concentrations of HA-PANBs were incubated with foam cells for 30 min; and (b and c) quantitative results; *: *P* ＜ 0.05, **: *P* ＜ 0.01.

The green fluorescence intensity of the 10^6^ group transfected with HA-PANBs for 30 m was 40.33 ± 1.1%, and the red fluorescence intensity was 0.067 ± 1.7%. The green fluorescence intensity of the 10^7^ group was 41.22 ± 1.3%, and the red fluorescence intensity was 41.22 ± 1.3%. The green fluorescence intensity of the 10^8^ group was 47.26 ± 1.9% and the red fluorescence intensity was 8.762 ± 0.9%, as shown in Appendix Material Figure A1.

**Figure A2:**
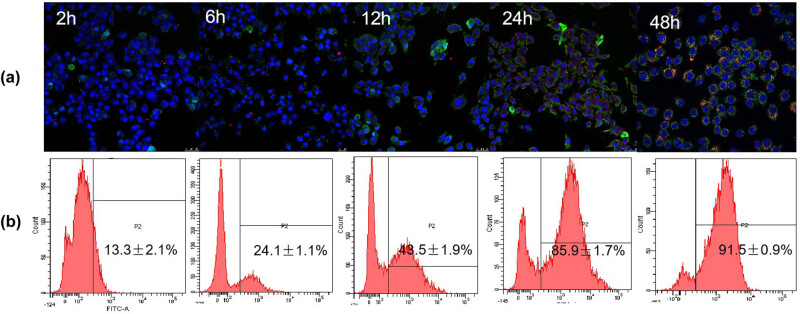
Laser confocal microscopy observation of HA-PANBs targeting *in vitro* at different times. (a) Representative images of laser confocal microscopy of different targeting conditions at different time points and (b) flow cytometric analysis of cell survival under different treatment conditions at indicated time points.

As shown in Appendix Material Figure A2, the binding of HA-PANBs to foam cells increased with the prolonged transfection time. At 48 h, the adsorption to cells of both AF-488 labeled PM1 and Cy3-labeled ANM33 reached an optimal level, as indicated by yellow colocalization. Flow cytometry showed that the binding rate of ANM33 was 91.5 ± 0.9%.

**Figure 7:**
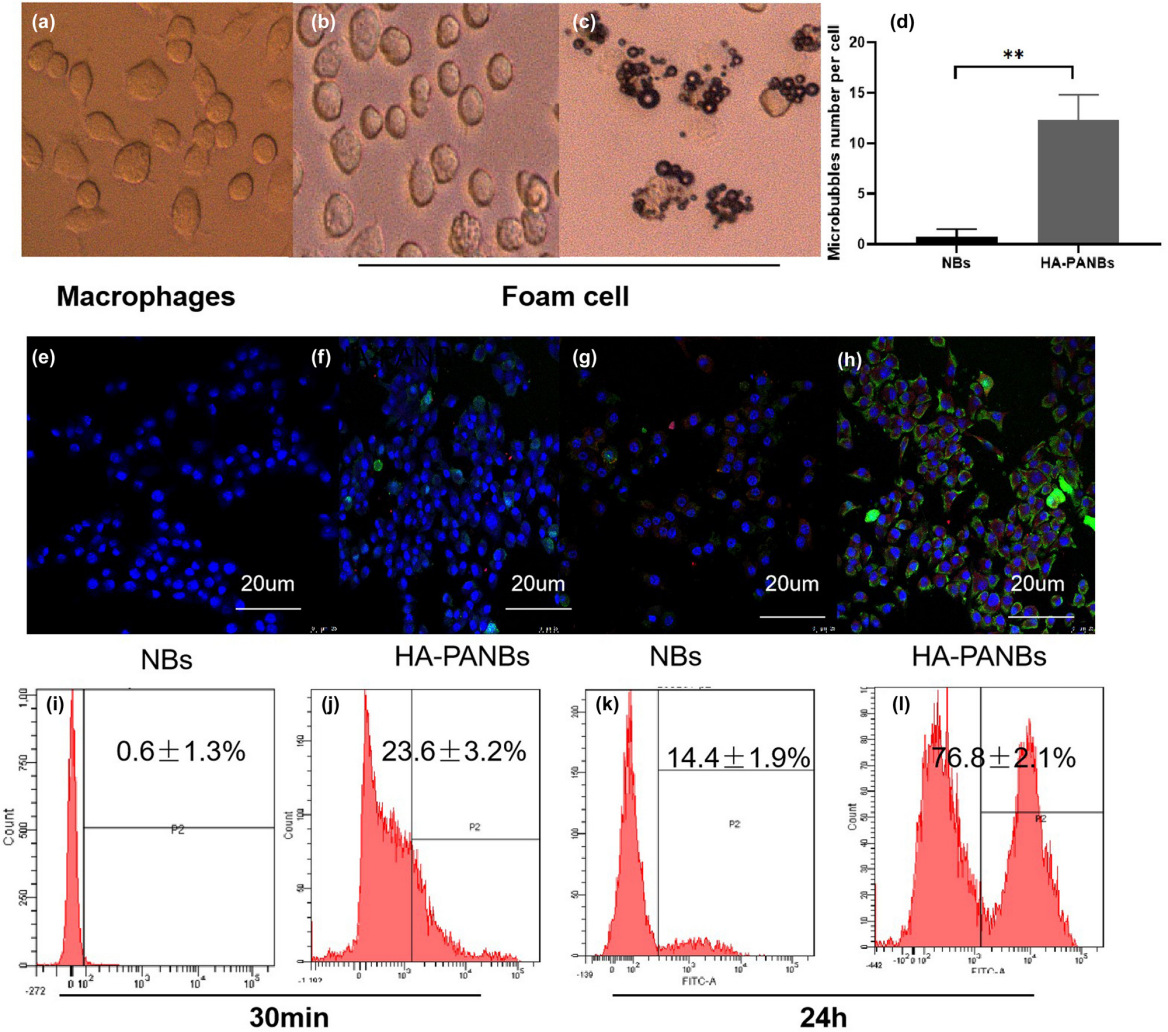
*In vitro* targeting of HA-PANBs. (a) Representative microscopic images of Mφ; (b) representative microscopic images of foam cells; (c) a large number of HA-PANBs targeting foam cells; (d) quantitative analysis of cell targeting; **P < 0.01; (e–h) laser confocal microscopy images of NBs and HA-PANBs targeting foam cells *in vitro* at different times; (i and l) flow cytometric analysis of NBs and HA-PANBs at different times.

The number of microbubbles around each foam cell in the HA-PANBs group was 14.7 ± 1.67, which was significantly higher than that in the NBs group (2.13 ± 0.57) (*P* < 0.05). The release rates for different groups are 0.6 ± 1.3% for NB and 23.6 ± 3.2% for HA-PANBs after 30 min, and 14.4 ± 1.9% for NB and 76.8 ± 2.1% for HA-PANBs after 24 h. The binding of HA-PANBs to foam cells is significantly different from those of non-target microvesicles and significantly higher than that of the NB group (*P* < 0.001) (Figure 7).

### 3.8 *In vitro* release of dual-targeted lipid microbubbles

**Figure 8:**
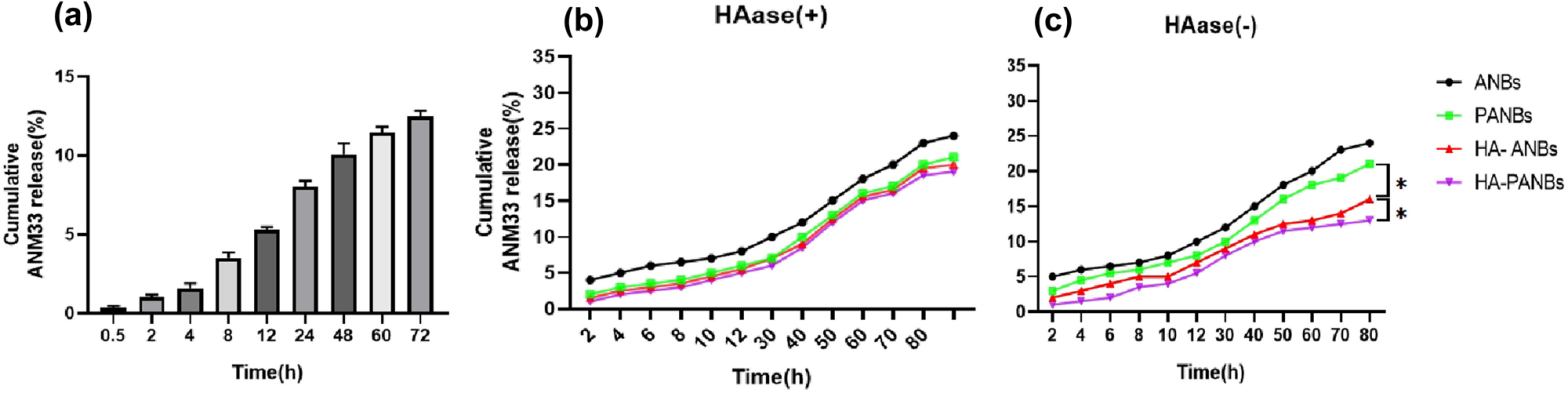
*In vitro* release of ANM33 in the presence and absence of HAase. (a) ANM33 release in HA-PANBs group within 72 h without HAase; (b) ANM33 release in the presence of HAase; and (c) ANM33 release profile in the absence of HAase. *: *P* < 0.05.

Figure 8 shows the schematics of ANM33 release from different microbubbles in the presence and absence of HAase. In the presence of HAase, the release rates from ANBs and PANBs within 72 h were approximately 23.19 ± 1.91% and 22.00 ± 1.71%, respectively. In the absence of HAase, the release rate from HA-PANBs was approximately 16.46 ± 1.57% after 72 h. In the case of HAase-catalyzed degradation, the ANM33 release rate from HA-PANBs was much higher (20.58% ± 1.43), which was almost at the same level as HA-ANBs and PANBs with or without HAase.

### 3.9 RT-PCR situation

**Figure 9:**
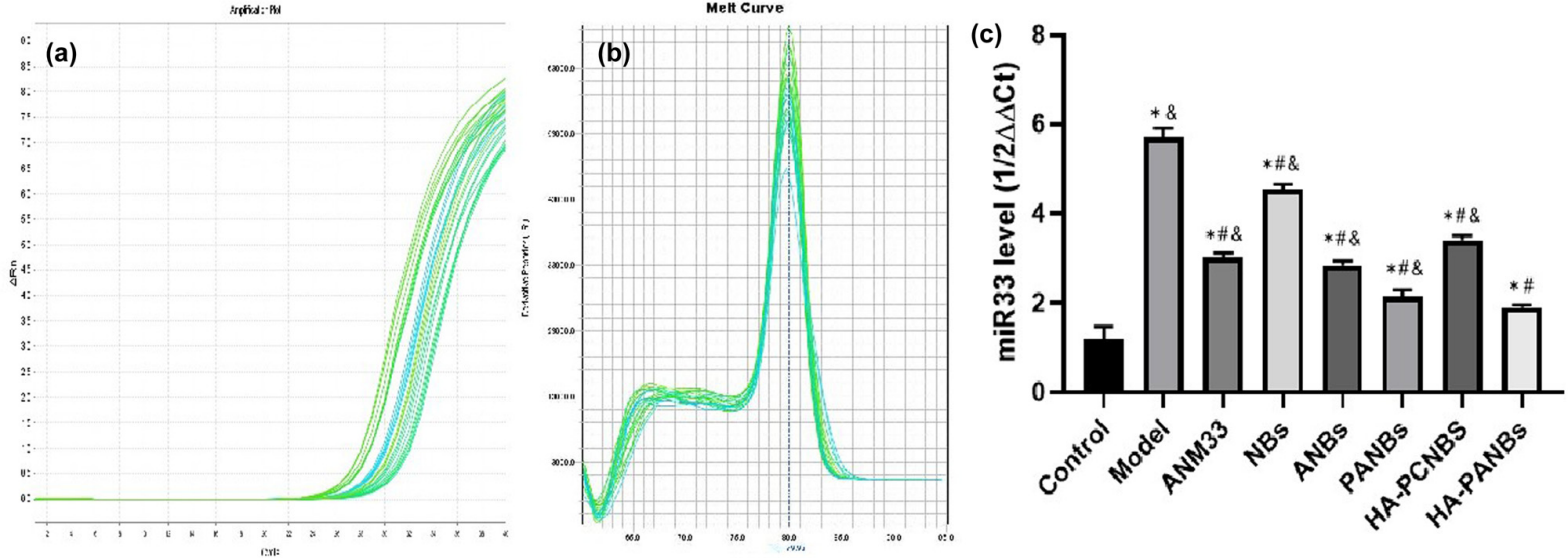
Effects of different interventions on miR33 expression in RAW264.7 cells and foam cell models. (a) Amplification curve; (b) dissolution curve; (c) quantitative results; *: *P* < 0.01 vs control group, #: *P* < 0.05 vs model group, &: *P* < 0.05 vs HA-PANBs group.

As shown in Figure 9, the quantitative RT-PCR shows the expression fold change of miR-33: control group 1.50 ± 0.9; model group 5.75 ± 1.1; ANM33 group 3.01 ± 2.3; NBs group: 4.55 ± 0.7; ANBs group 2.82 ± 1.1; PANBs group 2.03 ± 1.9; HA-PCNBs group 3.40 ± 0.6; HA-PANBs group 1.93 ± 2.3.

## 4 Discussion

We explored the optimal loading amount and release conditions of HA-PANBs. Our results indicate the following: (i) HA-PANBs efficiently delivers the ANM33 drug load into cells to exert its RNA silencing effect in a continuous manner. (ii) When using HA-PANBs at 12 mg/mL for 48 h, a higher release rate and drug efficacy were observed. (iii) It may represent a novel gene therapy approach to atherosclerosis (AS), making it an ideal ultrasound contrast agent.

It should be emphasized that accurate target adhesion is the basis of ultrasound molecular imaging. Furthermore, we showed that a large number of dual-target HA-PANBs microbubbles clustered around the foam cells but only a few with NB microbubbles. The HA-PANBs microbubbles also weakly adhere to Mφ, possibly due to non-specific endocytosis of Mφ. Quantitatively, the number of HA-PANBs adhering to foam cells was 6.9 times more than that of NBs. Meantime, more fluorescently labeled PM1 (green) and ANM33 (red) signals were associated with HA-PANBs than with NBs. After co-incubating with foam cells for 24 h, the release rate of targeted vesicles was 5.3 times more than that of non-targeted vesicles. Although a small number of cells in the NB group expressed red fluorescence peripherally under the fluorescence microscope, the flow cytometry revealed a cellular fluorescence carry-over rate of 14.4 ± 1.9%. The cellular transfection rate of dual-targeted lipid microvesicles reached about 76.8 ± 2.1%, which was five times higher than that of non-targeted vesicles (Figure 7). Guided by the results of HA-PANBs efficiently bound to foam cells, phased targeting strategy may be the direct reason for higher targeting rate: (i) the “invisible” HA coating might behave very similar to the hydrophilic polyethylene glycol [19], effectively shielding the internal lipid and drug contents from ineffective phagocytosis and uptake by the liver receptors, and (ii) HA-PANBs could target various biological barriers to Mφ through a multi-stage targeting mechanism.

Indeed, several previous studies have validated that the HA-coating may hinder the release of the therapeutic agent from HA-PANBs due to its potential in forming hydrophilic matrices around nanoparticles [20]. However, compared with the previous research, the present study, to the best of our knowledge, is the first attempt to examine the effect of dual-target microbubble loaded with ANM33 in foam cells. We found that ANM33 was continuously released from NBs, ANBs, PANBs, HA-CNBs, and HA-PANBs in the absence of HAase. Of note, compared with CNBs, HA-PANBs showed slower drug diffusion, probably due to a larger surface coverage by HA. In addition, there were no significant differences in the release pattern between NBs and ANBs in the presence and absence of HAase. When the enzyme treatment removed the HA coating from HA-PANBs and HA-CNBs, a higher drug release was observed, similar to the level of PANBs. This finding implies that both HA-PANBs and HA-CNBs are highly susceptible to HAase activity, subsequently exposing the encapsulated ANM33. As was our expectation, compared with microbubbles without the HA coating, the dissolution and drug release rates of HA-coated microbubbles were steadier, confirming that HA is better for lipid microbubble protection.

To further determine the optimal dose and release conditions of HA-PANBs, we concentrated with respect on the cellular phagocytic capacity with prolonged release time. Notably, the concentration of intracellular ANM33 gradually increased with increase in the time. The results showed, when 12 mg of HA-PANBs per 10^7^ cell for 48 h, a better foam cell targeting were observed than for 24 h. Laser confocal microscopy and flow cytometry results in Figure A1 from supplement materials provide us with a reasonable evidence to this result. Indeed, Bousoik et al. [21] have found that bivalent CD44 antibodies can promote Mφ-mediated phagocytosis. Rho is vital for phagocytosis of apoptotic cells by bone marrow-derived Mφ in mice [22,23], and given that the first phase of wrapping HA on the surface of our microvesicles for foam cell targeting resulted in an effective increase in targeting efficiency, it is reasonable to expect that it is due to the interaction of HA with CD44 expressed at endothelial cells that activates the Rho family in Mφ and drives its phagocytic process. This provides a new idea for HA to activate the intracellular Rho family after binding to the extracellular domain of CD44 on the surface of Mφ at the site of inflammation [24,25].

Guided by the results of RT-PCR situation, continuous and efficient targeted dissolution and release are the direct reasons for such recovery of the deregulation of miR-33 in HA-PANBs group: (1) The highest expression of miR-33 in the model group were consistent with the expectations. (2) The efficiency of single-target groups was increased compared with the model group, indicating that the target selection was reasonable; (3) The ANM33 group and ANBs group showed similar results, with not much of a reduction; (4) The HA-PANBs group obtained the ideal silencing effect. Our data are consistent with Kai and Xiaoqiu [26] for enhanced intracellular delivery of nanoparticles and provide the basis for further investigation of function and metabolic changes.

Cytotoxicity assays, including *in vitro* cell activity assays and *in vitro* hemolysis assays, were also performed in this study and the above results provide ample evidence of the safety of HA-PANBs. Of note, the material DSPE-PEG2000 can be dispersed in water to form micelles, and its critical micelle concentration is lower than that of surfactants [27,28]. Therefore, it is more suitable for the preparation of long-term nanomaterials, so as to prolong the circulation time in the blood and increase the chance of entering pathological tissues. Moreover, this experiment exploited the co-culture cell model referring to a study by Zhang et al. [29]. Our data showed that CD44 was significantly expressed in HUVECs of the experimental group (Figure 3), turning on early inflammatory responses of AS, which confirmed that the addition of TNF-α accelerated the recruitment of inflammatory cells. Additionally, the study by Tanzer et al. [30] has shown that TNF-α induces cell death (apoptosis and necrosis) similarly as reported by Li et al. [31]. On comparing with the control group, microbubble treatment groups did not exhibit any significant advancements in disease progression, possibly by suppressing the expression of miR-33 in these cells.

## 5 Conclusion

The results of the present study revealed that the dual-target ultrasound microbubble contrast agent HA-PANBs can effectively target the foam cells for RNA silencing. Moreover, we highlight that when HA-PANBs are used at 12 mg/mL per 10^7^ cells for 48 h, a higher release rate and drug efficacy were observed. In summary, we report a promising strategy for cardiovascular diseases, that exploits the providential advantages of AS therapy and multi-stage targeted delivery system.
